# The prevalence and genotype distribution of rotavirus A infection among children with acute gastroenteritis in Kunming, China

**DOI:** 10.1007/s00705-016-3102-6

**Published:** 2016-10-07

**Authors:** Ziqin Dian, Mao Fan, Binghui Wang, Yue Feng, Hao Ji, Shuwei Dong, A-Mei Zhang, Li Liu, Hua Niu, Xueshan Xia

**Affiliations:** 1 Faculty of Environmental Science and Engineering, Faculty of Life Science and Technology, Kunming University of Science and Technology, 727 Jingming South Road, Kunming, 650500 Yunnan China; 2 Department of Clinical Laboratory, First People’s Hospital of Yunnan Province, Kunming, 650032 Yunnan China; 3 Department of Clinical Laboratory, Kunming Children’s Hospital, Kunming, 650034 Yunnan China; 4 Department of Information Center, First People’s Hospital of Yunnan province, Kunming, 650032 Yunnan China

**Keywords:** Rotavirus A, Children, Gastroenteritis, Epidemiology, Genotype

## Abstract

The aim of this study was to determine the prevalence of rotavirus A (RVA) infections in children from Kunming, China, and the RVA genotypes present. A total of 16,311 children with acute gastroenteritis were recruited for the study, and 33.1 % (5,394/16,311) were RVA positive. Children under 24 months of age were more susceptible to RVA infection, with an infection rate of 87.4 % (4,712/5,394). The most prevalent genotype was G9P[8] (85/107, 79.4 %), which showed high sequence similarity to G9P[8] strains from other regions of China and neighbouring countries, but not to the licensed vaccine strain LLR. These findings should be useful for the prevention of RVA infections.

**Electronic supplementary material:**

The online version of this article (doi:10.1007/s00705-016-3102-6) contains supplementary material, which is available to authorized users.

Rotaviruses are important pathogens that cause acute gastroenteritis in both humans and animals. Rotavirus infections may lead to severe dehydration, electrolyte disorders, and acid-base disturbances. According to WHO estimates, the number of deaths of infected children worldwide in 2013 was 215,000 [1]. Rotaviruses belong to the family *Reoviridae*, whose members are characterized by a wheel-shaped capsid structure [2]. The virion consists of a triple-layered protein capsid containing 11 segments of dsRNA encoding six structural proteins (VP1-VP4, VP6, and VP7) and five or six nonstructural proteins (NSP1-NSP6) [3]. Rotaviruses have been classified into different genotypes based on the sequences of VP7 (G-type antigen) and VP4 (P-type antigen, which is cleaved into VP5* and VP8* by trypsin). In the last 30 years, at least 27 G and 37 P genotypes have been reported [4], and combinations of G1, G2, G3, G4, G9, and G12 with P[4], P[6], and P[8] have been found in humans [5]. Based on the serological characterization of VP6, rotaviruses have been classified into eight different groups (A-H). Among these, group A (RVA) is the most important pathogen in human children [6].

In China, the RVA infection rates in children range from 28 % to 65 %, and the hospitalization rate is 30-50 % [7, 8]. The city of Kunming is located in the southwestern region of China, with an annual average temperature 15 °C and a permanent resident population of 5,505,031. The population is composed of numerous ethnicities, and the economic and health status of the region is relatively poor. To date, few studies have reported molecular epidemiological data for RVA infections in Kunming [9]. In order to monitor the epidemiology of RVA and to estimate the effectiveness of the vaccine that is permitted for use in this area, the prevalence of RVA infection and genotypes among children with acute gastroenteritis were determined.

From February 2015 to January 2016, a total of 16,311 children under the age of 14 years with acute gastroenteritis were recruited from two representative hospitals (First People’s Hospital of Yunnan Province and Kunming Children’s Hospital). Demographic information was recorded during sampling. RVA antigens were detected using a Rapid Chromatographic RotaA Diagnostic Kit (Wantai, Beijing, CHN). RVA-positive faecal specimens were diluted 1:10 with PBS (Vircell, ESP, pH7.2) and centrifuged (8,000 rpm, 30 min). Then, the supernatants were stored at -80 °C. Subsequently, the viral genome was extracted from the supernatants using a TIANamp Virus RNA Kit (Tiangen Biotech, Beijing, CHN). RVA infection was confirmed by RT-PCR with VP6 primer [10] (Table S1).

In the present study, 120 samples were randomly selected from 5,394 RVA-positive specimens. Genotyping was performed using RT-nested PCR (RT-nPCR) with VP4 and VP7 primers (Table S1) [10, 11]. PCR products were purified using an Agarose Gel DNA Extraction Kit (Takara, Dalian, CHN) for commercial sequencing (Tsingke, Kunming, CHN). Sequences of VP7 and VP4 were submitted to GenBank under the accession numbers KX033584-KX033690 and KX033691-KX033797, respectively.

All sequences of VP7 and VP4 used in this study were aligned using the integrated Clustal X 1.83 programme, and neighbour-joining (NJ) trees were constructed in MEGA 6.0, using the Kimura 2-parameter model with gamma distribution and invariant sites as described previously [12]. The nodal reliability of the NJ trees was assessed by bootstrap (BS) analysis with 1000 pseudo-replicates; values below 70 % were excluded as non-significant. The percentage of nucleotide sequence identity was calculated using an online tool (http://www.genome.jp/tools/clustalw/).

Of the faecal samples collected from the 16,311 recruited children with acute gastroenteritis, 5,394 samples were found to be RVA positive, indicating an infection rate of 33.1 %. RVA infections are known to occur at a lower rate in spring and summer, with an increased incidence in late autumn. In the present study, a higher occurrence of RVA infections was detected from November 2015 to January 2016, with a peak infection rate of up to 71.5 % in November. However, the infection rate decreased to 6 %-8 % from July to September 2015 (Table S2), suggesting that the RVA infection rate was low and that gastroenteritis during this period was mainly caused by enterobacteria and enteric viruses, such as astrovirus, norovirus, and adenovirus [13]. Notably, children less than 2 years of age accounted for 87.4 % (4,712/5,394) of the total infections in the study period (Table S3). The infection rate showed an overall declining trend with increasing age after 36 months. Although RVA infections were also found in 5- to 14-year-old children, these accounted for only 2.0 % (110/5,394) of all positive samples (Table S3).

Among the 120 randomly selected RVA-positive specimens, VP7-encoding open reading frames (ORFs) and VP4 partial ORFs were successfully obtained from 107 (107/120, 89.2 %) and 112 (112/120, 93.3 %) samples, respectively. Mismatches between PCR primers and templates represent a possible reason for the failure of amplification of VP7 ORFs and partial VP4 ORFs. Five of 112 samples could not be genotyped with the VP7 primers, and this may be attributed to point mutations in the target regions [14], as has been reported previously [12].

The phylogenetic relationships of VP7 ORFs and VP4 partial ORFs to sequences from China and other countries were examined. Sequences of VP7 were separated into nine branches, and the present strains were classified under branches G1, G2, G3, G4, and G9. Eighty-nine G9 strains from this study were classified into two clusters with reference strains from China and neighbouring countries. Some strains formed several sub-clusters in one branch; however, km15060 was not related to other strains and formed an individual branch, which may imply the existence of a local endemic strain. Fifteen G2 strains formed two clusters with strains from China, Thailand, and Australia. The G3 and G4 branches consisted of strains from other regions of China and other countries; however, the G1 strain only clustered with foreign strains, which implied that this strain may have been imported into China (Fig. 1). Six genotypes, namely P[4], P[6], P[8], P[9], P[10], and P[14], were determined for VP4. Eighty-five P[8] strains with high nucleotide sequence similarity (98.6-100 % identity) formed cluster 1 with reference strains. Two strains, namely km15002 and km15009, were in cluster 2; the first clustered with strains from the USA, China and Japan, while the second clustered together with strains from Japan, Australia, Thailand, and Brazil. The latter group previously represented the most prevalent strains worldwide. Nineteen strains corresponding to the P[4] genotype were in the same cluster with foreign strains, and one P[6] strain was clustered with strains from China and other countries (Fig. 2).

**Fig. 1:**
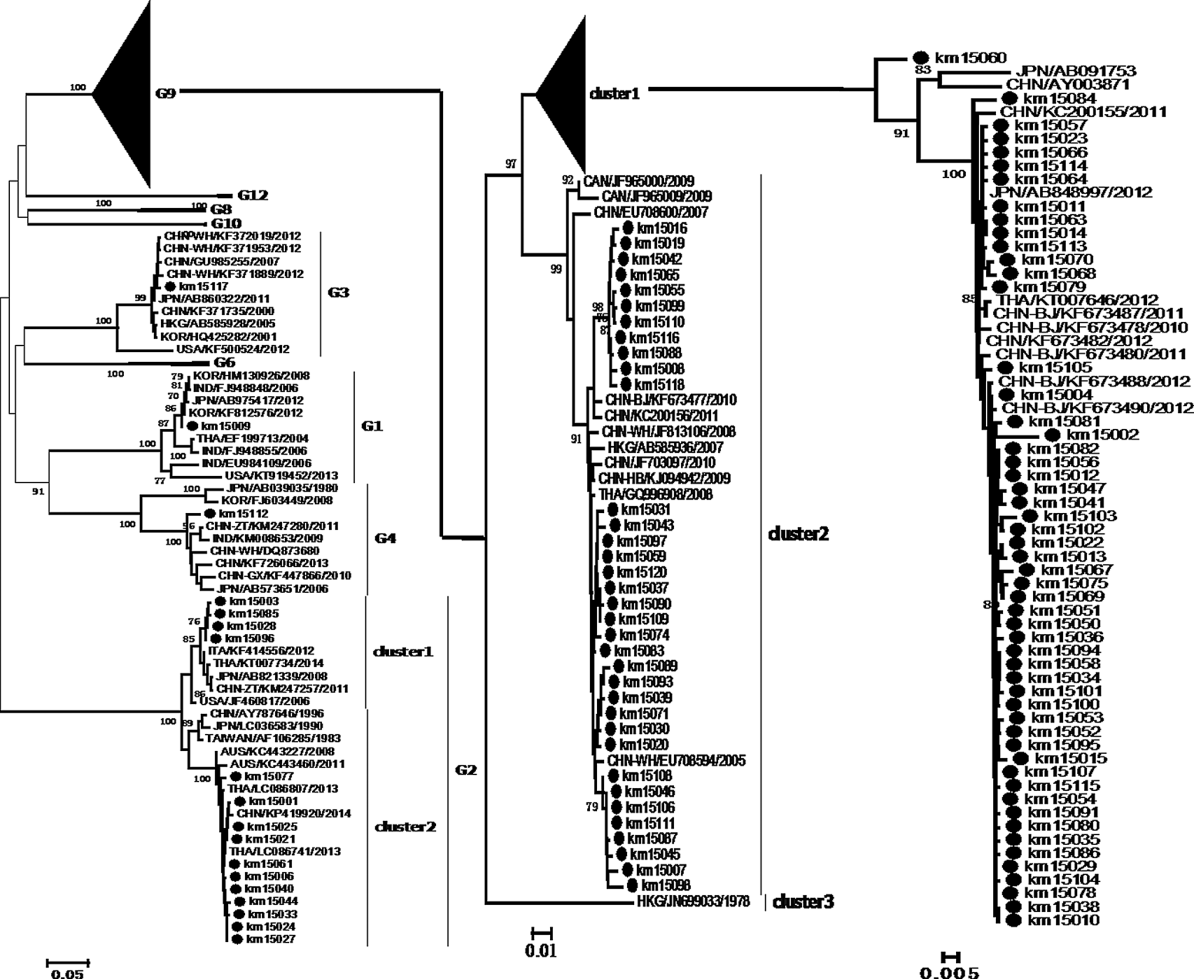
Phylogenetic trees of VP7 sequences. A neighbor-joining (NJ) tree was constructed using MEGA 6.0 software with the Kimura 2-parameter model with gamma distribution. Bootstrap values were determined for 1000 repetitions. Kunming strains are indicated by black dots. The sequences of reference strains were obtained from GenBank

**Fig. 2:**
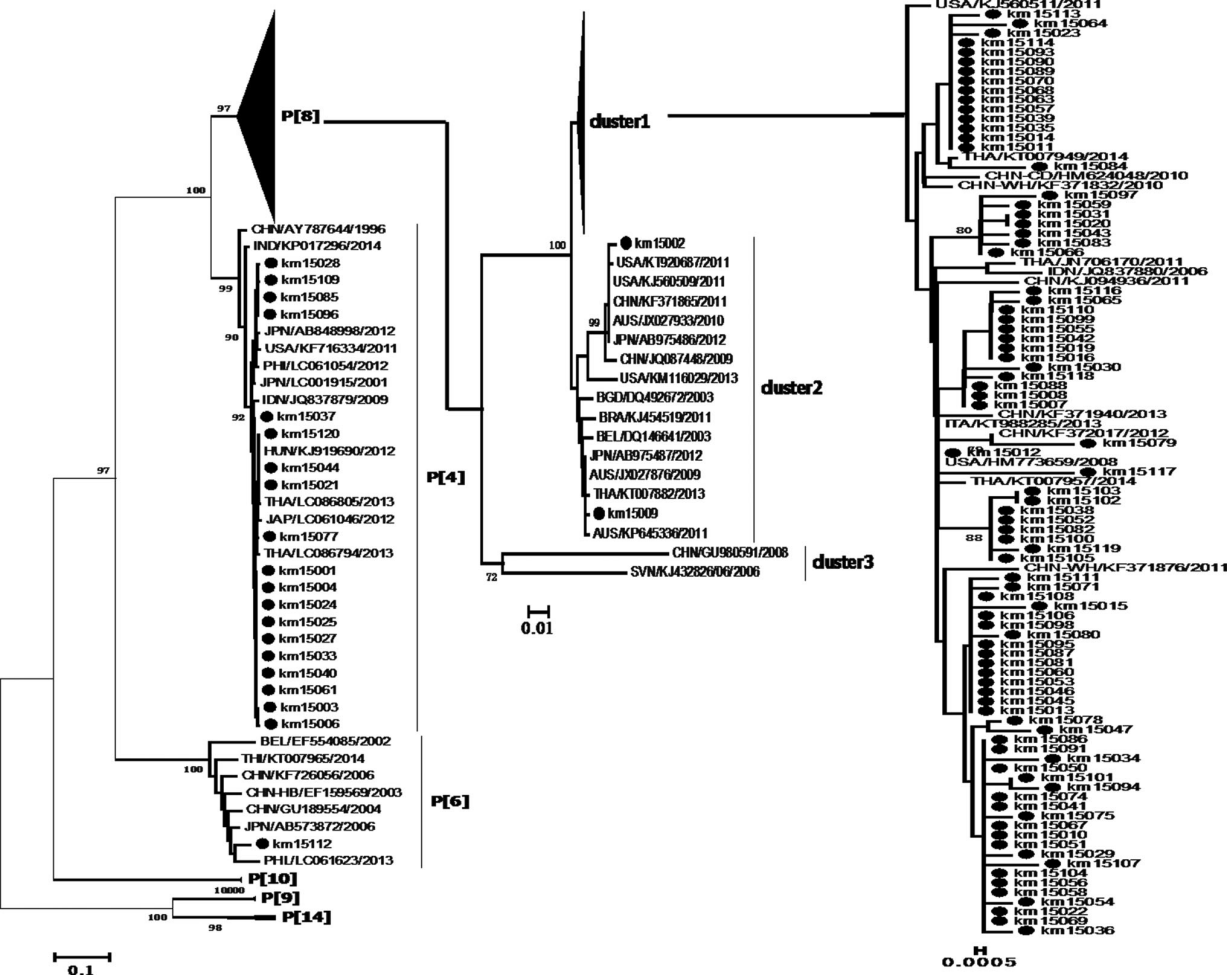
Phylogenetic trees of VP4 sequences. A neighbor-joining (NJ) tree was conducted using MEGA 6.0 software with the Kimura 2-parameter model with gamma distribution. Bootstrap values were determined for 1000 repetitions. Kunming strains are indicated by black dots. The sequences of reference strains were obtained from GenBank

Fifteen G2P[4] (15/107, 14.0 %) strains were distributed in five groups of children, mainly in the 13- to 24-month-old group (6/15, 40 %), the 25- to 36-month-old group (4/15, 26.7 %), and the 37- to 48-month-old group (3/15, 20 %), but not in the youngest group. This strain was considered to mainly infect older children [15] and accounted for 46.7 % (7/15) of infections in children 2 years of age. Four G9P[4] (4/107, 3.7 %) strains were detected in one child in the 0- to 12-month-old group, two in the 13- to 24-month-old group, and one in the 61- to 168-month-old group. The other genotypes included G1P[8] (1/107, 0.9 %), G3P[8] (1/107, 0.9 %), and G4P[6] (1/107, 0.9 %), which were detected in children 36, 13 and 20 months of age, respectively (Table 1). Although G1P[8] and G3P[8] strains were once widespread in China [5, 16], these strains were detected at a lower frequency in Kunming in the present study. These findings suggest that the G1-G4 strains, which represented the most prevalent genotypes worldwide in the past, are no longer the dominant strains in this region [17, 18].

**Table 1:** The RVA genotypes in children of different age groups

Genotype	0-12 M	13-24 M	25-36 M	37-48 M	49-60 M	61-168 M	Total
G9P[8]	36	38	5	4	1	1	85
G2P[4]	1	6	4	3	1	N	15
G9P[4]	1	2	N	N	N	1	4
G1P[8]	N	N	1	N	N	N	1
G3P[8]	N	1	N	N	N	N	1
G4P[6]	N	1	N	N	N	N	1

The total number of rotavirus genotyped for both G and P was 107. N, not detected

The distribution of the G9 strain is increasing worldwide. This genotype was first discovered in the USA in 1983 [19]; however, its prevalence was low for about a decade following its discovery. The distribution of the G9 strain increased worldwide after 2000 [20]. In China, the G9 strain was first identified in Yunnan province in 1998 [21]; only sporadic detections were reported in the following decade. By 2011, this genotype replaced the G1 and G3 strains and became the most prevalent genotype in Wuhan and Nanjing [16, 22]. In this study, G9P[8] was the most predominant genotype, accounting for 79.4 % (85/107) of all strains. The G9P[8] genotype was also prevalent in all age groups, especially in 0- to 12-month-olds (36/85, 42.4 %) and 13- to 24-month-olds (38/85, 44.7 %) (Table 1).

It has been suggested that vaccination programs should be mainly targeted at younger children [13]. The amino acid sequence similarity of the dominant G9P[8] strains in this study and three vaccine strains were analysed. Lanzhou lamb rotavirus vaccine (LLR), which is a G10P[15]-based monovalent live attenuated oral vaccine licensed in China, was used. The other two vaccines were Rotarix™ (G1P[8]) and RotaTeq™ (G1-G4P[8]), which are commonly used worldwide, but not in China. Amino acid residues on the surface of VP7 and VP4 that are responsible for neutralization were designated as 7-1a, 7-1b and 7-2, 8-1, 8-2, 8-3 and 8-4, respectively. By Clustal-W alignment of the VP7 amino acids of the G9P[8] strains to LLR, 14 amino acid (aa) substitutions were found in 28 aa (Table S4). In addition, alignments of the VP8* subunit sequences of G9P[8] strains to Rotarix™ and RotaTeq™ revealed that G9P[8] strains were more closely related to the latter, with four aa substitutions in eight aa. A low degree of homology was found between the VP8* subunit of G9P[8] and LLR, with 21 aa substitutions in 25 aa (Table S5). These findings suggest that these licensed vaccines may not be suitable for use in children in Kunming. LLR is licensed in China but is not included in national immunization programmes, and its effectiveness and safety are not known [23]. Effective vaccination programmes should be designed to reduce morbidity resulting from RVA infections.

The high frequency of G9 strains observed in this study implies that this genotype is widespread at least in Kunming, and it may be potentially transmitted to other regions, as an important port for routes to Southeast Asian countries is located in Yunnan province. It is not clear whether the use of existing vaccines has resulted in a reduction in the distribution of other genotypes or if mutations have led to the immunological escape of this genotype. Further studies investigating the molecular basis of the transmission advantage of G9 strains are required.

## Electronic supplementary material

Below is the link to the electronic supplementary material.

Supplementary material 1 (DOC 708 kb)
