# Supplementary material for: The prevalence and genotype distribution of rotavirus A infection among children with acute gastroenteritis in Kunming, China
Source: Arch Virol. 2016 Oct 7;162(1):281–5. doi: 10.1007/s00705-016-3102-6 (PMC5225225; doi:10.1007/s00705-016-3102-6)
Supplement: Supplementary file 1 — Supplementary material 1 (DOC 708 kb) [file 705_2016_3102_MOESM1_ESM.doc]

**The prevalence and genotypes distribution of Rotavirus A among children with acute gastroenteritis in Kunming, China.**

**Archives of Virology**

Ziqin Dian^1, 2+^, Mao Fan^3+^, Binghui Wang^1^, Yue Feng^1^, Hao Ji^4^, Shuwei Dong^1^, A-Mei Zhang^1^, Li Liu^1^, Hua Niu^2^, Xueshan Xia^1*^

1 Faculty of Environmental Science and Engineering, Faculty of Life Science and Technology, Kunming University of Science and Technology, Yunnan 650500, China

2 Department of Clinical laboratory, First People’s Hospital of Yunnan province, Yunnan 650032, China

3 Department of Clinical laboratory, Kunming Children’s Hospital, Yunnan 650034, China

4 Department of Information center, First People’s Hospital of Yunnan province, Yunnan 650032, China

^+^Ziqin Dian and Mao Fan contribute to the article equally

*Corresponding to: Xueshan Xia

Phone: 86-871-65920576

Fax: 86-871-65920576

*E-mail address: oliverxia2000@aliyun.com (Xueshan Xia)*

*Faculty of Life Science and Technology, Kunming University of Science and Technology, 727 Jingming South Road, Kunming 650500, China.*

**Table S1** Primers used in this study

| | Primers | nucleotide position | Sequences(5'-3') | Product(bp) | | --- | --- | --- | --- | | VP6-Fwd  VP6-Rev  VP4-WF  VP4-WR  VP4-NF  VP4-NR  VP7-Beg9  VP7-End9  VP7-NF  VP7-NR | 4-30  754-783  10-39  1116-1149  142-159  785-805  1--28  1036-1062  49-71  914-933 | TTTAAAACGAAGTCTTCRACATGGAKG  TAATTGGATTAAARAACCATGTAGTYGCKC  ATGGCTTCGCTCATTTATAGACARCTTCTC  ACTGARTTYARATTAGCTGCTAATGATCTRACAT  TATGCTCCAGTNAATTGG  ATTGCATTTCTTTCCATAATG  GGCTTTAAAAGAGAGAATTTCCGTCTGG  GGTCACATCATACAATTCTAATCTAAG  ATGTATGGTATTGAATATACCAC  AACTTGCCACCATTTTTTCC | 779  1139  663  1034  884 | |
| --- | --- | --- | --- | --- | --- | --- | --- | --- |

R represented A and G; K represented G and T; Y represented C and T; N represented A, T, G, and C.

**Table S2** The prevalence of gastroenteritis and RVA infection among children in Kunming from February 2015 to January 2016.

|  | Feb | Mar | Apr | May | Jun | Jul | Aug | Sep | Oct | Nov | Dec | Jan |
| --- | --- | --- | --- | --- | --- | --- | --- | --- | --- | --- | --- | --- |
| Gastroenteritis cases | 846 | 1406 | 1300 | 1525 | 1499 | 1333 | 1271 | 1105 | 1241 | 1868 | 1682 | 1235 |
| Positive RVA cases | 261 | 585 | 327 | 310 | 220 | 91 | 71 | 90 | 223 | 1336 | 1042 | 838 |
| Positive Rate (%) | 30.9 | 41.6 | 25.2 | 20.3 | 14.7 | 6.8 | 5.6 | 8.1 | 18.0 | 71.5 | 62.0 | 67.9 |

**Table S3** The results of RVA detection in different age and gender groups of children from March 2015 to February 2016

|  | RVA | 0-12M | 13-24M | 25-36M | 37-48M | 49-60M | 61-168M | Total |
| --- | --- | --- | --- | --- | --- | --- | --- | --- |
| Male  Female  Total | Positive  Negative  Positive  Negative | 1392  2771  914  2011  7088 | 1430  2913  976  1894  7213 | 221  413  151  293  1078 | 87  191  62  131  471 | 38  61  13  31  143 | 65  119  45  89  318 | 3233  6468  2161  4449  16311 |

**Table S4** Alignment of the amino acid residues defining the neutralization domains (designated as 7-1a, 7-1b and 7-2) of VP7 between the LLR and Chinese G9P[8] RVA strains detected in Kunming. Red indicates the residues different from LLR.

|  | **G9-VP7** | | | | | | | | | | | | | | | | | | | | | | | | | | | | | | |
| --- | --- | --- | --- | --- | --- | --- | --- | --- | --- | --- | --- | --- | --- | --- | --- | --- | --- | --- | --- | --- | --- | --- | --- | --- | --- | --- | --- | --- | --- | --- | --- |
|  | **7-1a** | | | | | | | | | | | | | |  | **7-1b** | | | | | |  | **7-2** | | | | | | | | |
| **HM800948/LLR/1985** | **87** | **91** | **94** | **96** | **97** | **98** | **99** | **100** | **104** | **123** | **125** | **129** | **130** |  |  | **201** | **211** | **212** | **213** | **238** | **242** |  | **143** | **145** | **146** | **147** | **148** | **190** | **217** | **221** | **264** |
|  | **I** | **T** | **N** | **N** | **E** | **W** | **T** | **S** | **Q** | **N** | **A** | **V** | **D** |  |  | **Q** | **N** | **T** | **G** | **D** | **T** |  | **R** | **N** | **S** | **S** | **L** | **S** | **E** | **A** | **G** |
| **CHN/km15002/G9P[8]** | **T** | **T** | **G** | **T** | **E** | **W** | **K** | **N** | **Q** | **D** | **T** | **I** | **D** |  |  | **Q** | **N** | **T** | **A** | **D** | **N** |  | **K** | **D** | **S** | **T** | **L** | **S** | **E** | **S** | **G** |
| **CHN/km15007/G9P[8]** | **T** | **T** | **G** | **A** | **E** | **W** | **K** | **D** | **Q** | **D** | **A** | **I** | **D** |  |  | **Q** | **N** | **T** | **A** | **D** | **N** |  | **K** | **D** | **S** | **T** | **L** | **S** | **E** | **S** | **G** |
| **CHN/km1508/G9P[8]** | **T** | **T** | **G** | **T** | **E** | **W** | **K** | **D** | **Q** | **D** | **A** | **I** | **D** |  |  | **Q** | **N** | **T** | **A** | **D** | **N** |  | **K** | **D** | **S** | **T** | **L** | **S** | **E** | **G** | **G** |
| **CHN/km15010/G9P[8]** | **T** | **T** | **G** | **T** | **E** | **W** | **K** | **N** | **Q** | **D** | **T** | **I** | **D** |  |  | **Q** | **N** | **T** | **A** | **D** | **N** |  | **K** | **D** | **S** | **T** | **L** | **S** | **E** | **S** | **G** |
| **CHN/km15011/G9P[8]** | **T** | **T** | **G** | **T** | **E** | **W** | **K** | **N** | **Q** | **D** | **A** | **I** | **D** |  |  | **Q** | **N** | **T** | **A** | **D** | **N** |  | **K** | **D** | **S** | **T** | **L** | **S** | **E** | **S** | **G** |
| **CHN/km15012/G9P[8]** | **T** | **T** | **G** | **T** | **E** | **W** | **K** | **N** | **Q** | **D** | **T** | **I** | **D** |  |  | **Q** | **N** | **T** | **A** | **D** | **N** |  | **K** | **D** | **S** | **T** | **L** | **S** | **E** | **S** | **G** |
| **CHN/km15013/G9P[8]** | **T** | **T** | **G** | **T** | **E** | **W** | **K** | **N** | **Q** | **D** | **T** | **I** | **D** |  |  | **Q** | **N** | **T** | **A** | **D** | **N** |  | **K** | **D** | **S** | **T** | **L** | **S** | **E** | **S** | **G** |
| **CHN/km15014/G9P[8]** | **T** | **T** | **G** | **T** | **E** | **W** | **K** | **N** | **Q** | **D** | **A** | **I** | **D** |  |  | **Q** | **N** | **T** | **A** | **D** | **N** |  | **K** | **D** | **S** | **T** | **L** | **S** | **E** | **S** | **G** |
| **CHN/km15015/G9P[8]** | **T** | **T** | **G** | **T** | **E** | **W** | **K** | **N** | **Q** | **D** | **T** | **I** | **D** |  |  | **Q** | **N** | **T** | **A** | **D** | **N** |  | **K** | **D** | **S** | **T** | **L** | **S** | **E** | **S** | **G** |
| **CHN/km15016/G9P[8]** | **T** | **T** | **G** | **T** | **E** | **W** | **K** | **D** | **Q** | **D** | **A** | **I** | **D** |  |  | **Q** | **N** | **T** | **A** | **D** | **N** |  | **K** | **D** | **S** | **T** | **L** | **S** | **E** | **G** | **G** |
| **CHN/km15019/G9P[8]** | **T** | **T** | **G** | **T** | **E** | **W** | **K** | **D** | **Q** | **D** | **A** | **I** | **D** |  |  | **Q** | **N** | **T** | **A** | **D** | **N** |  | **K** | **D** | **S** | **T** | **L** | **S** | **E** | **G** | **G** |
| **CHN/km15020/G9P[8]** | **T** | **T** | **G** | **T** | **E** | **W** | **K** | **D** | **Q** | **D** | **A** | **I** | **D** |  |  | **Q** | **N** | **T** | **A** | **D** | **N** |  | **K** | **D** | **S** | **T** | **L** | **S** | **E** | **S** | **G** |
| **CHN/km15022/G9P[8]** | **T** | **T** | **G** | **T** | **E** | **W** | **K** | **N** | **Q** | **D** | **T** | **I** | **D** |  |  | **Q** | **N** | **T** | **A** | **D** | **N** |  | **K** | **D** | **S** | **T** | **L** | **S** | **E** | **S** | **G** |
| **CHN/km15023/G9P[8]** | **T** | **T** | **G** | **T** | **E** | **W** | **K** | **N** | **Q** | **D** | **A** | **I** | **D** |  |  | **Q** | **N** | **T** | **A** | **D** | **N** |  | **K** | **D** | **S** | **T** | **L** | **S** | **E** | **S** | **G** |
| **CHN/km15029/G9P[8]** | **T** | **T** | **G** | **T** | **E** | **W** | **K** | **N** | **Q** | **D** | **T** | **I** | **D** |  |  | **Q** | **N** | **T** | **A** | **D** | **N** |  | **K** | **D** | **S** | **T** | **L** | **S** | **E** | **S** | **G** |
| **CHN/km15030/G9P[8]** | **T** | **T** | **G** | **T** | **E** | **W** | **K** | **D** | **Q** | **D** | **A** | **I** | **D** |  |  | **Q** | **N** | **T** | **A** | **D** | **N** |  | **K** | **D** | **S** | **T** | **L** | **S** | **E** | **S** | **G** |
| **CHN/km15031/G9P[8]** | **T** | **T** | **G** | **T** | **E** | **W** | **K** | **D** | **Q** | **D** | **A** | **I** | **D** |  |  | **Q** | **N** | **T** | **A** | **D** | **N** |  | **K** | **D** | **S** | **T** | **L** | **S** | **E** | **S** | **G** |
| **CHN/km15034/G9P[8]** | **T** | **T** | **G** | **T** | **E** | **W** | **K** | **N** | **Q** | **D** | **T** | **I** | **D** |  |  | **Q** | **N** | **T** | **A** | **D** | **N** |  | **K** | **D** | **S** | **T** | **L** | **S** | **E** | **S** | **G** |
| **CHN/km15035/G9P[8]** | **T** | **T** | **G** | **T** | **E** | **W** | **K** | **N** | **Q** | **D** | **T** | **I** | **D** |  |  | **Q** | **N** | **T** | **A** | **D** | **N** |  | **K** | **D** | **S** | **T** | **L** | **S** | **E** | **S** | **G** |
| **CHN/km15036/G9P[8]** | **T** | **T** | **G** | **T** | **E** | **W** | **K** | **N** | **Q** | **D** | **T** | **I** | **D** |  |  | **Q** | **N** | **T** | **A** | **D** | **N** |  | **K** | **D** | **S** | **T** | **L** | **S** | **E** | **S** | **G** |
| **CHN/km15038/G9P[8]** | **T** | **T** | **G** | **T** | **E** | **W** | **K** | **N** | **Q** | **D** | **T** | **I** | **D** |  |  | **Q** | **N** | **T** | **A** | **D** | **N** |  | **K** | **D** | **S** | **T** | **L** | **S** | **E** | **S** | **G** |
| **CHN/km15039/G9P[8]** | **T** | **T** | **G** | **T** | **E** | **W** | **K** | **D** | **Q** | **D** | **A** | **I** | **D** |  |  | **Q** | **N** | **T** | **A** | **D** | **N** |  | **K** | **D** | **S** | **T** | **L** | **S** | **E** | **S** | **G** |
| **CHN/km15041/G9P[8]** | **T** | **T** | **G** | **T** | **E** | **W** | **K** | **N** | **Q** | **D** | **T** | **I** | **D** |  |  | **Q** | **N** | **T** | **A** | **D** | **N** |  | **K** | **D** | **S** | **T** | **L** | **S** | **E** | **S** | **G** |
| **CHN/km15042/G9P[8]** | **T** | **T** | **G** | **T** | **E** | **W** | **K** | **D** | **Q** | **D** | **A** | **I** | **D** |  |  | **Q** | **N** | **T** | **A** | **D** | **N** |  | **K** | **D** | **S** | **T** | **L** | **S** | **E** | **G** | **G** |
| **CHN/km15043/G9P[8]** | **T** | **T** | **G** | **T** | **E** | **W** | **K** | **D** | **Q** | **D** | **A** | **I** | **D** |  |  | **Q** | **N** | **T** | **A** | **D** | **N** |  | **K** | **D** | **S** | **T** | **L** | **S** | **E** | **S** | **G** |
| **CHN/km15045/G9P[8]** | **T** | **T** | **G** | **T** | **E** | **W** | **K** | **D** | **Q** | **D** | **A** | **I** | **D** |  |  | **Q** | **N** | **T** | **A** | **D** | **N** |  | **K** | **D** | **S** | **T** | **L** | **S** | **E** | **S** | **G** |
| **CHN/km15046/G9P[8]** | **T** | **T** | **G** | **T** | **E** | **W** | **K** | **D** | **Q** | **D** | **A** | **I** | **D** |  |  | **Q** | **N** | **T** | **A** | **D** | **N** |  | **K** | **D** | **S** | **T** | **L** | **S** | **E** | **S** | **G** |
| **CHN/km15047/G9P[8]** | **T** | **T** | **G** | **T** | **E** | **W** | **K** | **N** | **Q** | **D** | **T** | **I** | **D** |  |  | **Q** | **N** | **T** | **A** | **D** | **N** |  | **K** | **D** | **S** | **T** | **L** | **S** | **E** | **S** | **G** |
| **CHN/km15050/G9P[8]** | **T** | **T** | **G** | **T** | **E** | **W** | **K** | **N** | **Q** | **D** | **T** | **I** | **D** |  |  | **Q** | **N** | **T** | **A** | **D** | **N** |  | **K** | **D** | **S** | **T** | **L** | **S** | **E** | **S** | **G** |
| **CHN/km15051/G9P[8]** | **T** | **T** | **G** | **T** | **E** | **W** | **K** | **N** | **Q** | **D** | **T** | **I** | **D** |  |  | **Q** | **N** | **T** | **A** | **D** | **N** |  | **K** | **D** | **S** | **T** | **L** | **S** | **E** | **S** | **G** |
| **CHN/km15052/G9P[8]** | **T** | **T** | **G** | **T** | **E** | **W** | **K** | **N** | **Q** | **D** | **T** | **I** | **D** |  |  | **Q** | **N** | **T** | **A** | **D** | **N** |  | **K** | **D** | **S** | **T** | **L** | **S** | **E** | **S** | **G** |
| **CHN/km15053/G9P[8]** | **T** | **T** | **G** | **T** | **E** | **W** | **K** | **N** | **Q** | **D** | **T** | **I** | **D** |  |  | **Q** | **N** | **T** | **A** | **D** | **N** |  | **K** | **D** | **S** | **T** | **L** | **S** | **E** | **S** | **G** |
| **CHN/km15054/G9P[8]** | **T** | **T** | **G** | **T** | **E** | **W** | **K** | **N** | **Q** | **D** | **T** | **I** | **D** |  |  | **Q** | **N** | **T** | **A** | **D** | **N** |  | **K** | **D** | **S** | **T** | **L** | **S** | **E** | **S** | **G** |
| **CHN/km15055/G9P[8]** | **T** | **T** | **G** | **T** | **E** | **W** | **K** | **D** | **Q** | **D** | **A** | **I** | **D** |  |  | **Q** | **N** | **T** | **A** | **D** | **N** |  | **K** | **D** | **S** | **T** | **L** | **S** | **E** | **G** | **G** |
| **CHN/km15056/G9P[8]** | **T** | **T** | **G** | **T** | **E** | **W** | **K** | **N** | **Q** | **D** | **T** | **I** | **D** |  |  | **Q** | **N** | **T** | **A** | **D** | **N** |  | **K** | **D** | **S** | **T** | **L** | **S** | **E** | **S** | **G** |
| **CHN/km15057/G9P[8]** | **T** | **T** | **G** | **T** | **E** | **W** | **K** | **N** | **Q** | **D** | **A** | **I** | **D** |  |  | **Q** | **N** | **T** | **A** | **D** | **N** |  | **K** | **D** | **S** | **T** | **L** | **S** | **E** | **S** | **G** |
| **CHN/km15058/G9P[8]** | **T** | **T** | **G** | **T** | **E** | **W** | **K** | **N** | **Q** | **D** | **T** | **I** | **D** |  |  | **Q** | **N** | **T** | **A** | **D** | **N** |  | **K** | **D** | **S** | **T** | **L** | **S** | **E** | **S** | **G** |
| **CHN/km15059/G9P[8]** | **T** | **T** | **G** | **T** | **E** | **W** | **K** | **D** | **Q** | **D** | **A** | **I** | **D** |  |  | **Q** | **N** | **T** | **A** | **D** | **N** |  | **K** | **D** | **S** | **T** | **L** | **S** | **E** | **S** | **G** |
| **CHN/km15060/G9P[8]** | **T** | **T** | **G** | **T** | **E** | **W** | **K** | **D** | **Q** | **D** | **A** | **I** | **D** |  |  | **Q** | **N** | **T** | **A** | **D** | **N** |  | **K** | **D** | **S** | **T** | **L** | **S** | **E** | **S** | **G** |
| **CHN/km15063/G9P[8]** | **T** | **T** | **G** | **T** | **E** | **W** | **K** | **N** | **Q** | **D** | **A** | **I** | **D** |  |  | **Q** | **N** | **T** | **A** | **D** | **N** |  | **K** | **D** | **S** | **T** | **L** | **S** | **E** | **S** | **G** |
| **CHN/km15064/G9P[8]** | **T** | **T** | **G** | **T** | **E** | **W** | **K** | **N** | **Q** | **D** | **A** | **I** | **D** |  |  | **Q** | **N** | **T** | **A** | **D** | **N** |  | **K** | **D** | **S** | **T** | **L** | **S** | **E** | **S** | **G** |
| **CHN/km15065/G9P[8]** | **T** | **T** | **G** | **T** | **E** | **W** | **K** | **D** | **Q** | **D** | **A** | **I** | **D** |  |  | **Q** | **N** | **T** | **A** | **D** | **N** |  | **K** | **D** | **S** | **T** | **L** | **S** | **E** | **G** | **G** |
| **CHN/km15066/G9P[8]** | **T** | **T** | **G** | **T** | **E** | **W** | **K** | **N** | **Q** | **D** | **A** | **I** | **D** |  |  | **Q** | **N** | **T** | **A** | **D** | **N** |  | **K** | **D** | **S** | **T** | **L** | **S** | **E** | **S** | **G** |
| **CHN/km15067/G9P[8]** | **T** | **T** | **G** | **T** | **E** | **W** | **K** | **N** | **Q** | **D** | **T** | **I** | **D** |  |  | **Q** | **N** | **T** | **A** | **D** | **N** |  | **K** | **D** | **S** | **T** | **L** | **S** | **E** | **S** | **G** |
| **CHN/km15068/G9P[8]** | **T** | **T** | **G** | **T** | **E** | **W** | **K** | **N** | **Q** | **D** | **A** | **I** | **D** |  |  | **Q** | **N** | **T** | **A** | **D** | **N** |  | **K** | **D** | **S** | **T** | **L** | **S** | **E** | **S** | **G** |
| **CHN/km15069/G9P[8]** | **T** | **T** | **G** | **T** | **E** | **W** | **K** | **N** | **Q** | **D** | **T** | **I** | **D** |  |  | **Q** | **N** | **T** | **A** | **D** | **N** |  | **K** | **D** | **S** | **T** | **L** | **S** | **E** | **S** | **G** |
| **CHN/km15070/G9P[8]** | **T** | **T** | **G** | **T** | **E** | **W** | **K** | **N** | **Q** | **D** | **A** | **I** | **D** |  |  | **Q** | **N** | **T** | **A** | **D** | **N** |  | **K** | **D** | **S** | **T** | **L** | **S** | **E** | **S** | **G** |
| **CHN/km15071/G9P[8]** | **T** | **T** | **G** | **T** | **E** | **W** | **K** | **D** | **Q** | **D** | **A** | **I** | **D** |  |  | **Q** | **N** | **T** | **A** | **D** | **N** |  | **K** | **D** | **S** | **T** | **L** | **S** | **E** | **S** | **G** |
| **CHN/km15074/G9P[8]** | **T** | **T** | **G** | **T** | **E** | **W** | **K** | **D** | **Q** | **D** | **A** | **I** | **D** |  |  | **Q** | **N** | **T** | **A** | **D** | **N** |  | **K** | **D** | **S** | **T** | **L** | **S** | **E** | **S** | **G** |
| **CHN/km15075/G9P[8]** | **T** | **T** | **G** | **T** | **E** | **W** | **K** | **N** | **Q** | **D** | **T** | **I** | **D** |  |  | **Q** | **N** | **T** | **A** | **D** | **N** |  | **K** | **D** | **S** | **T** | **L** | **S** | **E** | **S** | **G** |
| **CHN/km15078/G9P[8]** | **T** | **T** | **G** | **T** | **E** | **W** | **K** | **N** | **Q** | **D** | **T** | **I** | **D** |  |  | **Q** | **N** | **T** | **A** | **D** | **N** |  | **K** | **D** | **S** | **T** | **L** | **S** | **E** | **S** | **G** |
| **CHN/km15079/G9P[8]** | **T** | **T** | **G** | **T** | **E** | **W** | **K** | **N** | **Q** | **D** | **A** | **I** | **D** |  |  | **Q** | **N** | **T** | **A** | **D** | **N** |  | **K** | **D** | **S** | **T** | **L** | **S** | **E** | **S** | **G** |
| **CHN/km15080/G9P[8]** | **T** | **T** | **G** | **T** | **E** | **W** | **K** | **N** | **Q** | **D** | **T** | **I** | **D** |  |  | **Q** | **N** | **T** | **A** | **D** | **N** |  | **K** | **D** | **S** | **T** | **L** | **S** | **E** | **S** | **G** |
| **CHN/km15081/G9P[8]** | **T** | **T** | **G** | **T** | **E** | **W** | **K** | **N** | **Q** | **D** | **T** | **I** | **D** |  |  | **Q** | **N** | **T** | **A** | **D** | **N** |  | **K** | **D** | **S** | **T** | **L** | **S** | **E** | **S** | **G** |
| **CHN/km15082/G9P[8]** | **T** | **T** | **G** | **T** | **E** | **W** | **K** | **N** | **Q** | **D** | **T** | **I** | **D** |  |  | **Q** | **N** | **T** | **A** | **D** | **N** |  | **K** | **D** | **S** | **T** | **L** | **S** | **E** | **S** | **G** |
| **CHN/km15083/G9P[8]** | **T** | **T** | **G** | **T** | **E** | **W** | **K** | **D** | **Q** | **D** | **A** | **I** | **D** |  |  | **Q** | **N** | **T** | **A** | **D** | **N** |  | **K** | **D** | **S** | **T** | **L** | **S** | **E** | **S** | **G** |
| **CHN/km15084/G9P[8]** | **T** | **T** | **G** | **T** | **E** | **W** | **K** | **N** | **Q** | **D** | **A** | **I** | **D** |  |  | **Q** | **N** | **T** | **A** | **D** | **N** |  | **K** | **D** | **S** | **T** | **L** | **S** | **E** | **S** | **G** |
| **CHN/km15086/G9P[8]** | **T** | **T** | **G** | **T** | **E** | **W** | **K** | **N** | **Q** | **D** | **T** | **I** | **D** |  |  | **Q** | **N** | **T** | **A** | **D** | **N** |  | **K** | **D** | **S** | **T** | **L** | **S** | **E** | **S** | **G** |
| **CHN/km15087/G9P[8]** | **T** | **T** | **G** | **T** | **E** | **W** | **K** | **D** | **Q** | **D** | **A** | **I** | **G** |  |  | **Q** | **N** | **T** | **A** | **D** | **N** |  | **K** | **D** | **S** | **T** | **L** | **S** | **E** | **S** | **G** |
| **CHN/km15088/G9P[8]** | **T** | **T** | **G** | **T** | **E** | **W** | **K** | **D** | **Q** | **D** | **A** | **I** | **D** |  |  | **Q** | **N** | **T** | **A** | **D** | **N** |  | **K** | **D** | **S** | **T** | **L** | **S** | **E** | **G** | **G** |
| **CHN/km15089/G9P[8]** | **T** | **T** | **G** | **T** | **E** | **W** | **K** | **D** | **Q** | **D** | **A** | **I** | **D** |  |  | **Q** | **N** | **T** | **A** | **D** | **N** |  | **K** | **D** | **S** | **T** | **L** | **S** | **E** | **S** | **G** |
| **CHN/km15090/G9P[8]** | **T** | **T** | **G** | **T** | **E** | **W** | **K** | **D** | **Q** | **D** | **A** | **I** | **D** |  |  | **Q** | **N** | **T** | **A** | **D** | **N** |  | **K** | **D** | **S** | **T** | **L** | **S** | **E** | **S** | **G** |
| **CHN/km15091/G9P[8]** | **T** | **T** | **G** | **T** | **E** | **W** | **K** | **N** | **Q** | **D** | **T** | **I** | **D** |  |  | **Q** | **N** | **T** | **A** | **D** | **N** |  | **K** | **D** | **S** | **T** | **L** | **S** | **E** | **S** | **G** |
| **CHN/km15093/G9P[8]** | **T** | **T** | **G** | **T** | **E** | **W** | **K** | **D** | **Q** | **D** | **A** | **I** | **D** |  |  | **Q** | **N** | **T** | **A** | **D** | **N** |  | **K** | **D** | **S** | **T** | **L** | **S** | **E** | **S** | **G** |
| **CHN/km15094/G9P[8]** | **T** | **T** | **G** | **T** | **E** | **W** | **K** | **N** | **Q** | **D** | **T** | **I** | **D** |  |  | **Q** | **N** | **T** | **A** | **D** | **N** |  | **K** | **D** | **S** | **T** | **L** | **S** | **E** | **S** | **G** |
| **CHN/km15095/G9P[8]** | **T** | **T** | **G** | **T** | **E** | **W** | **K** | **N** | **Q** | **D** | **T** | **I** | **D** |  |  | **Q** | **N** | **T** | **A** | **D** | **N** |  | **K** | **D** | **S** | **T** | **L** | **S** | **E** | **S** | **G** |
| **CHN/km15097/G9P[8]** | **T** | **T** | **G** | **T** | **E** | **W** | **K** | **D** | **Q** | **D** | **A** | **I** | **D** |  |  | **Q** | **N** | **T** | **A** | **D** | **N** |  | **K** | **D** | **S** | **T** | **L** | **S** | **E** | **S** | **G** |
| **CHN/km15098/G9P[8]** | **T** | **T** | **G** | **T** | **E** | **W** | **K** | **D** | **Q** | **D** | **A** | **I** | **D** |  |  | **Q** | **N** | **T** | **A** | **D** | **N** |  | **K** | **D** | **S** | **T** | **L** | **S** | **E** | **S** | **G** |
| **CHN/km15099/G9P[8]** | **T** | **T** | **G** | **T** | **E** | **W** | **K** | **D** | **Q** | **D** | **A** | **I** | **D** |  |  | **Q** | **N** | **T** | **A** | **D** | **N** |  | **K** | **D** | **S** | **T** | **L** | **S** | **E** | **G** | **G** |
| **CHN/km15100/G9P[8]** | **T** | **T** | **G** | **T** | **E** | **W** | **K** | **N** | **Q** | **D** | **T** | **I** | **D** |  |  | **Q** | **N** | **T** | **A** | **D** | **N** |  | **K** | **D** | **S** | **T** | **L** | **S** | **E** | **S** | **G** |
| **CHN/km15101/G9P[8]** | **T** | **T** | **G** | **T** | **E** | **W** | **K** | **N** | **Q** | **D** | **T** | **I** | **D** |  |  | **Q** | **N** | **T** | **A** | **D** | **N** |  | **K** | **D** | **S** | **T** | **L** | **S** | **E** | **S** | **G** |
| **CHN/km15102/G9P[8]** | **T** | **T** | **G** | **T** | **E** | **W** | **K** | **N** | **Q** | **D** | **T** | **I** | **D** |  |  | **Q** | **N** | **T** | **A** | **D** | **N** |  | **K** | **D** | **S** | **T** | **L** | **S** | **E** | **S** | **G** |
| **CHN/km15103/G9P[8]** | **T** | **T** | **G** | **T** | **E** | **W** | **K** | **N** | **Q** | **D** | **T** | **I** | **D** |  |  | **Q** | **N** | **T** | **A** | **D** | **N** |  | **K** | **D** | **S** | **T** | **L** | **S** | **E** | **S** | **G** |
| **CHN/km15104/G9P[8]** | **T** | **T** | **G** | **T** | **E** | **W** | **K** | **N** | **Q** | **D** | **T** | **I** | **D** |  |  | **Q** | **N** | **T** | **A** | **D** | **N** |  | **K** | **D** | **S** | **T** | **L** | **S** | **E** | **S** | **G** |
| **CHN/km15105/G9P[8]** | **T** | **T** | **G** | **T** | **E** | **W** | **K** | **N** | **Q** | **D** | **A** | **I** | **D** |  |  | **Q** | **N** | **T** | **A** | **D** | **N** |  | **K** | **D** | **S** | **T** | **L** | **S** | **E** | **S** | **G** |
| **CHN/km15106/G9P[8]** | **T** | **T** | **G** | **T** | **E** | **W** | **K** | **D** | **Q** | **D** | **A** | **I** | **D** |  |  | **Q** | **N** | **T** | **A** | **D** | **N** |  | **K** | **D** | **S** | **T** | **L** | **S** | **E** | **S** | **G** |
| **CHN/km15107/G9P[8]** | **T** | **T** | **G** | **T** | **E** | **W** | **K** | **N** | **Q** | **D** | **T** | **I** | **D** |  |  | **Q** | **N** | **T** | **A** | **D** | **N** |  | **K** | **D** | **S** | **T** | **L** | **S** | **E** | **S** | **G** |
| **CHN/km15108/G9P[8]** | **T** | **T** | **G** | **T** | **E** | **W** | **K** | **D** | **Q** | **D** | **A** | **I** | **D** |  |  | **Q** | **N** | **T** | **A** | **D** | **N** |  | **K** | **D** | **S** | **T** | **L** | **S** | **E** | **S** | **G** |
| **CHN/km15110/G9P[8]** | **T** | **T** | **G** | **T** | **E** | **W** | **K** | **D** | **Q** | **D** | **A** | **I** | **D** |  |  | **Q** | **N** | **T** | **A** | **D** | **N** |  | **K** | **D** | **S** | **T** | **L** | **S** | **E** | **G** | **G** |
| **CHN/km15111/G9P[8]** | **T** | **T** | **G** | **T** | **E** | **W** | **K** | **D** | **Q** | **D** | **A** | **I** | **D** |  |  | **Q** | **N** | **T** | **A** | **D** | **N** |  | **K** | **D** | **S** | **T** | **L** | **S** | **E** | **S** | **G** |
| **CHN/km15113/G9P[8]** | **T** | **T** | **G** | **T** | **E** | **W** | **K** | **N** | **Q** | **D** | **A** | **I** | **D** |  |  | **Q** | **N** | **T** | **A** | **D** | **N** |  | **K** | **D** | **S** | **T** | **L** | **S** | **E** | **S** | **G** |
| **CHN/km15114/G9P[8]** | **T** | **T** | **G** | **T** | **E** | **W** | **K** | **N** | **Q** | **D** | **A** | **I** | **D** |  |  | **Q** | **N** | **T** | **A** | **D** | **N** |  | **K** | **D** | **S** | **T** | **L** | **S** | **E** | **S** | **G** |
| **CHN/km15116/G9P[8]** | **T** | **T** | **G** | **T** | **E** | **W** | **K** | **N** | **Q** | **D** | **T** | **I** | **D** |  |  | **Q** | **N** | **T** | **A** | **D** | **N** |  | **K** | **D** | **S** | **T** | **L** | **S** | **E** | **S** | **G** |
| **CHN/km15118/G9P[8]** | **T** | **T** | **G** | **T** | **E** | **W** | **K** | **D** | **Q** | **D** | **A** | **I** | **D** |  |  | **Q** | **N** | **T** | **A** | **D** | **N** |  | **K** | **D** | **S** | **T** | **L** | **S** | **E** | **G** | **G** |

**Table S5** Alignment of the amino acid residues corresponding to those defining the VP4 neutralization domains (designated as 8-1, 8-2, 8-3 and 8-4) in the VP8* subunit between the Rotarix™ , RotaTeq™ , LLR and Chinese G9P[8] RVA strains detected in Kunming. Red indicates the residues different from those of both Rotarix™, RotaTeq™ and LLR. Yellow indicates the residues identical to those of both Rotarix™, RotaTeq™ but different from LLR. Blue indicates the residues identical to those of RotaTeq™, but different from those of Rotarix™ and LLR.

|  | **P[8]-VP8*** | | | | | | | | | | | | | | | | | | | | | | | | | | | |
| --- | --- | --- | --- | --- | --- | --- | --- | --- | --- | --- | --- | --- | --- | --- | --- | --- | --- | --- | --- | --- | --- | --- | --- | --- | --- | --- | --- | --- |
|  | **8-1** | | | | | | | | | | |  | **8-2** | |  | **8-3** | | | | | | | | |  | **8-4** | | |
|  | **101** | **146** | **148** | **150** | **188** | **190** | **192** | **193** | **194** | **195** | **196** |  | **180** | **183** |  | **114** | **115** | **116** | **117** | **126** | **132** | **133** | **134** | **136** |  | **88** | **89** | **90** |
| **JX943612.2/Rotarix/G1P8** | **D** | **S** | **S** | **N** | **S** | **S** | **A** | **N** | **L** | **N** | **N** |  | **E** | **R** |  | **N** | **P** | **V** | **D** | **S** | **S** | **N** | **D** | **N** |  | **N** | **T** | **N** |
| **GU565044/RotaTeq/G6P1A8**  **JQ013506/LLR/1982/G10P[15]** | **D**  **D** | **S**  **T** | **S**  **S** | **N**  **W** | **S**  **Y** | **N**  **S** | **A**  **T** | **N**  **N** | **L**  **Y** | **N**  **D** | **D**  **S** |  | **E**  **E** | **R**  **N** |  | **N**  **P** | **P**  **E** | **V**  **T** | **D**  **T** | **N**  **S** | **R**  **K** | **N**  **P** | **D**  **I** | **D**  **N** |  | **N**  **T** | **T**  **S** | **N**  **E** |
| **CHN/km15002/G9P[8]** | **D** | **G** | **S** | **N** | **S** | **N** | **A** | **N** | **L** | **N** | **G** |  | **E** | **R** |  | **D** | **P** | **V** | **D** | **N** | **R** | **N** | **D** | **D** |  | **N** | **T** | **N** |
| **CHN/km15007/G9P[8]** | **D** | **G** | **S** | **S** | **S** | **N** | **A** | **N** | **L** | **N** | **G** |  | **E** | **R** |  | **D** | **P** | **V** | **D** | **N** | **R** | **N** | **D** | **D** |  | **N** | **T** | **N** |
| **CHN/km15008/G9P[8]** | **D** | **G** | **S** | **S** | **S** | **N** | **A** | **N** | **L** | **N** | **G** |  | **E** | **R** |  | **D** | **P** | **V** | **D** | **N** | **R** | **N** | **D** | **D** |  | **N** | **T** | **N** |
| **CHN/km15010/G9P[8]** | **D** | **G** | **S** | **S** | **S** | **N** | **A** | **N** | **L** | **N** | **G** |  | **E** | **R** |  | **D** | **P** | **V** | **D** | **N** | **R** | **N** | **D** | **D** |  | **N** | **T** | **N** |
| **CHN/km15011/G9P[8]** | **D** | **G** | **S** | **S** | **S** | **N** | **A** | **N** | **L** | **N** | **G** |  | **E** | **R** |  | **D** | **P** | **V** | **D** | **N** | **R** | **N** | **D** | **D** |  | **N** | **T** | **N** |
| **CHN/km15012/G9P[8]** | **D** | **G** | **S** | **S** | **S** | **N** | **A** | **N** | **L** | **N** | **G** |  | **E** | **R** |  | **D** | **P** | **V** | **D** | **N** | **R** | **N** | **D** | **D** |  | **N** | **T** | **N** |
| **CHN/km15013/G9P[8]** | **D** | **G** | **S** | **S** | **S** | **N** | **A** | **N** | **L** | **N** | **G** |  | **E** | **R** |  | **D** | **P** | **V** | **D** | **N** | **R** | **N** | **D** | **D** |  | **N** | **T** | **N** |
| **CHN/km15014/G9P[8]** | **D** | **G** | **S** | **S** | **S** | **N** | **A** | **N** | **L** | **N** | **G** |  | **E** | **R** |  | **D** | **P** | **V** | **D** | **N** | **R** | **N** | **D** | **D** |  | **N** | **T** | **N** |
| **CHN/km15015/G9P[8]** | **D** | **G** | **S** | **S** | **S** | **N** | **A** | **N** | **L** | **N** | **G** |  | **E** | **R** |  | **D** | **P** | **V** | **D** | **N** | **R** | **N** | **D** | **D** |  | **N** | **T** | **N** |
| **CHN/km15016/G9P[8]** | **D** | **G** | **S** | **S** | **S** | **N** | **A** | **N** | **L** | **N** | **G** |  | **E** | **R** |  | **D** | **P** | **V** | **D** | **N** | **R** | **N** | **D** | **D** |  | **N** | **T** | **N** |
| **CHN/km15019/G9P[8]** | **D** | **G** | **S** | **S** | **S** | **N** | **A** | **N** | **L** | **N** | **G** |  | **E** | **R** |  | **D** | **P** | **V** | **D** | **N** | **R** | **N** | **D** | **D** |  | **N** | **T** | **N** |
| **CHN/km15020/G9P[8]** | **D** | **G** | **S** | **S** | **S** | **N** | **A** | **N** | **L** | **N** | **G** |  | **E** | **R** |  | **D** | **P** | **V** | **D** | **N** | **R** | **N** | **D** | **D** |  | **N** | **T** | **N** |
| **CHN/km15022/G9P[8]** | **D** | **G** | **S** | **S** | **S** | **N** | **A** | **N** | **L** | **N** | **G** |  | **E** | **R** |  | **D** | **P** | **V** | **D** | **N** | **R** | **N** | **D** | **D** |  | **N** | **T** | **N** |
| **CHN/km15023/G9P[8]** | **D** | **G** | **S** | **S** | **S** | **N** | **A** | **N** | **L** | **N** | **G** |  | **E** | **R** |  | **D** | **P** | **V** | **D** | **N** | **R** | **N** | **D** | **D** |  | **N** | **T** | **N** |
| **CHN/km15029/G9P[8]** | **D** | **G** | **S** | **S** | **S** | **N** | **A** | **N** | **L** | **N** | **G** |  | **E** | **R** |  | **D** | **P** | **V** | **D** | **N** | **R** | **N** | **D** | **D** |  | **N** | **T** | **N** |
| **CHN/km15030/G9P[8]** | **D** | **G** | **S** | **S** | **S** | **N** | **A** | **N** | **L** | **N** | **G** |  | **E** | **R** |  | **D** | **P** | **V** | **D** | **N** | **R** | **N** | **D** | **D** |  | **N** | **T** | **N** |
| **CHN/km15031/G9P[8]** | **D** | **G** | **S** | **S** | **S** | **N** | **A** | **N** | **L** | **N** | **G** |  | **E** | **R** |  | **D** | **P** | **V** | **D** | **N** | **R** | **N** | **D** | **D** |  | **N** | **T** | **N** |
| **CHN/km15034/G9P[8]** | **D** | **G** | **S** | **S** | **S** | **N** | **A** | **N** | **L** | **N** | **G** |  | **E** | **R** |  | **D** | **P** | **V** | **D** | **N** | **R** | **N** | **D** | **D** |  | **N** | **T** | **N** |
| **CHN/km15035/G9P[8]** | **D** | **G** | **S** | **S** | **S** | **N** | **A** | **N** | **L** | **N** | **G** |  | **E** | **R** |  | **D** | **P** | **V** | **D** | **N** | **R** | **N** | **D** | **D** |  | **N** | **T** | **N** |
| **CHN/km15036/G9P[8]** | **D** | **G** | **S** | **S** | **S** | **N** | **A** | **N** | **L** | **N** | **G** |  | **E** | **R** |  | **D** | **P** | **V** | **D** | **N** | **R** | **N** | **D** | **D** |  | **N** | **T** | **N** |
| **CHN/km15038/G9P[8]** | **D** | **G** | **S** | **S** | **S** | **N** | **A** | **N** | **L** | **N** | **G** |  | **E** | **R** |  | **D** | **P** | **V** | **D** | **N** | **R** | **N** | **D** | **D** |  | **N** | **T** | **N** |
| **CHN/km15039/G9P[8]** | **D** | **G** | **S** | **S** | **S** | **N** | **A** | **N** | **L** | **N** | **G** |  | **E** | **R** |  | **D** | **P** | **V** | **D** | **N** | **R** | **N** | **D** | **D** |  | **N** | **T** | **N** |
| **CHN/km15041/G9P[8]** | **D** | **G** | **S** | **S** | **S** | **N** | **A** | **N** | **L** | **N** | **G** |  | **E** | **R** |  | **D** | **P** | **V** | **D** | **N** | **R** | **N** | **D** | **D** |  | **N** | **T** | **N** |
| **CHN/km15042/G9P[8]** | **D** | **G** | **S** | **S** | **S** | **N** | **A** | **N** | **L** | **N** | **G** |  | **E** | **R** |  | **D** | **P** | **V** | **D** | **N** | **R** | **N** | **D** | **D** |  | **N** | **T** | **N** |
| **CHN/km15043/G9P[8]** | **D** | **G** | **S** | **S** | **S** | **N** | **A** | **N** | **L** | **N** | **G** |  | **E** | **R** |  | **D** | **P** | **V** | **D** | **N** | **R** | **N** | **D** | **D** |  | **N** | **T** | **N** |
| **CHN/km15045/G9P[8]** | **D** | **G** | **S** | **S** | **S** | **N** | **A** | **N** | **L** | **N** | **G** |  | **E** | **R** |  | **D** | **P** | **V** | **D** | **N** | **R** | **N** | **D** | **D** |  | **N** | **T** | **N** |
| **CHN/km15046/G9P[8]** | **D** | **G** | **S** | **S** | **S** | **N** | **A** | **N** | **L** | **N** | **G** |  | **E** | **R** |  | **D** | **P** | **V** | **D** | **N** | **R** | **N** | **D** | **D** |  | **N** | **T** | **N** |
| **CHN/km15047/G9P[8]** | **D** | **G** | **S** | **S** | **S** | **N** | **A** | **N** | **L** | **N** | **G** |  | **E** | **R** |  | **D** | **P** | **V** | **D** | **N** | **R** | **N** | **D** | **D** |  | **N** | **T** | **N** |
| **CHN/km15050/G9P[8]** | **D** | **G** | **S** | **S** | **S** | **N** | **A** | **N** | **L** | **N** | **G** |  | **E** | **R** |  | **D** | **P** | **V** | **D** | **N** | **R** | **N** | **D** | **D** |  | **N** | **T** | **N** |
| **CHN/km15051/G9P[8]** | **D** | **G** | **S** | **S** | **S** | **N** | **A** | **N** | **L** | **N** | **G** |  | **E** | **R** |  | **D** | **P** | **V** | **D** | **N** | **R** | **N** | **D** | **D** |  | **N** | **T** | **N** |
| **CHN/km15052/G9P[8]** | **D** | **G** | **S** | **S** | **S** | **N** | **A** | **N** | **L** | **N** | **G** |  | **E** | **R** |  | **D** | **P** | **V** | **D** | **N** | **R** | **N** | **D** | **D** |  | **N** | **T** | **N** |
| **CHN/km15053/G9P[8]** | **D** | **G** | **S** | **S** | **S** | **N** | **A** | **N** | **L** | **N** | **G** |  | **E** | **R** |  | **D** | **P** | **V** | **D** | **N** | **R** | **N** | **D** | **D** |  | **N** | **T** | **N** |
| **CHN/km15054/G9P[8]** | **D** | **G** | **S** | **S** | **S** | **N** | **A** | **N** | **L** | **N** | **G** |  | **E** | **R** |  | **D** | **P** | **V** | **D** | **N** | **R** | **N** | **D** | **D** |  | **N** | **T** | **N** |
| **CHN/km15055/G9P[8]** | **D** | **G** | **S** | **S** | **S** | **N** | **A** | **N** | **L** | **N** | **G** |  | **E** | **R** |  | **D** | **P** | **V** | **D** | **N** | **R** | **N** | **D** | **D** |  | **N** | **T** | **N** |
| **CHN/km15056/G9P[8]** | **D** | **G** | **S** | **S** | **S** | **N** | **A** | **N** | **L** | **N** | **G** |  | **E** | **R** |  | **D** | **P** | **V** | **D** | **N** | **R** | **N** | **D** | **D** |  | **N** | **T** | **N** |
| **CHN/km15057/G9P[8]** | **D** | **G** | **S** | **S** | **S** | **N** | **A** | **N** | **L** | **N** | **G** |  | **E** | **R** |  | **D** | **P** | **V** | **D** | **N** | **R** | **N** | **D** | **D** |  | **N** | **T** | **N** |
| **CHN/km15058/G9P[8]** | **D** | **G** | **S** | **S** | **S** | **N** | **A** | **N** | **L** | **N** | **G** |  | **E** | **R** |  | **D** | **P** | **V** | **D** | **N** | **R** | **N** | **D** | **D** |  | **N** | **T** | **N** |
| **CHN/km15059/G9P[8]** | **D** | **G** | **S** | **S** | **S** | **N** | **A** | **N** | **L** | **N** | **G** |  | **E** | **R** |  | **D** | **P** | **V** | **D** | **N** | **R** | **N** | **D** | **D** |  | **N** | **T** | **N** |
| **CHN/km15060/G9P[8]** | **D** | **G** | **S** | **S** | **S** | **N** | **A** | **N** | **L** | **N** | **G** |  | **E** | **R** |  | **D** | **P** | **V** | **D** | **N** | **R** | **N** | **D** | **D** |  | **N** | **T** | **N** |
| **CHN/km15063/G9P[8]** | **D** | **G** | **S** | **S** | **S** | **N** | **A** | **N** | **L** | **N** | **G** |  | **E** | **R** |  | **D** | **P** | **V** | **D** | **N** | **R** | **N** | **D** | **D** |  | **N** | **T** | **N** |
| **CHN/km15064/G9P[8]** | **D** | **G** | **S** | **S** | **S** | **N** | **A** | **N** | **L** | **N** | **G** |  | **E** | **R** |  | **D** | **P** | **V** | **D** | **N** | **R** | **N** | **D** | **D** |  | **N** | **T** | **N** |
| **CHN/km15065/G9P[8]** | **D** | **G** | **S** | **S** | **S** | **N** | **A** | **N** | **L** | **N** | **G** |  | **E** | **R** |  | **D** | **P** | **V** | **D** | **N** | **R** | **N** | **D** | **D** |  | **N** | **T** | **N** |
| **CHN/km15066/G9P[8]** | **D** | **G** | **S** | **S** | **S** | **N** | **A** | **N** | **L** | **N** | **G** |  | **E** | **R** |  | **D** | **P** | **V** | **D** | **N** | **R** | **N** | **D** | **D** |  | **N** | **T** | **N** |
| **CHN/km15067/G9P[8]** | **D** | **G** | **S** | **S** | **S** | **N** | **A** | **N** | **L** | **N** | **G** |  | **E** | **R** |  | **D** | **P** | **V** | **D** | **N** | **R** | **N** | **D** | **D** |  | **N** | **T** | **N** |
| **CHN/km15068/G9P[8]** | **D** | **G** | **S** | **S** | **S** | **N** | **A** | **N** | **L** | **N** | **G** |  | **E** | **R** |  | **D** | **P** | **V** | **D** | **N** | **R** | **N** | **D** | **D** |  | **N** | **T** | **N** |
| **CHN/km15069/G9P[8]** | **D** | **G** | **S** | **S** | **S** | **N** | **A** | **N** | **L** | **N** | **G** |  | **E** | **R** |  | **D** | **P** | **V** | **D** | **N** | **R** | **N** | **D** | **D** |  | **N** | **T** | **N** |
| **CHN/km15070/G9P[8]** | **D** | **G** | **S** | **S** | **S** | **N** | **A** | **N** | **L** | **N** | **G** |  | **E** | **R** |  | **D** | **P** | **V** | **D** | **N** | **R** | **N** | **D** | **D** |  | **N** | **T** | **N** |
| **CHN/km15071/G9P[8]** | **D** | **G** | **S** | **S** | **S** | **N** | **A** | **N** | **L** | **N** | **G** |  | **E** | **R** |  | **D** | **P** | **V** | **D** | **N** | **R** | **N** | **D** | **D** |  | **N** | **T** | **N** |
| **CHN/km15074/G9P[8]** | **D** | **G** | **S** | **S** | **S** | **N** | **A** | **N** | **L** | **N** | **G** |  | **E** | **R** |  | **D** | **P** | **V** | **D** | **N** | **R** | **N** | **D** | **D** |  | **N** | **T** | **N** |
| **CHN/km15075/G9P[8]** | **D** | **G** | **S** | **S** | **S** | **N** | **A** | **N** | **L** | **N** | **G** |  | **E** | **R** |  | **D** | **P** | **V** | **D** | **N** | **R** | **N** | **D** | **D** |  | **D** | **T** | **N** |
| **CHN/km15078/G9P[8]** | **D** | **G** | **S** | **S** | **S** | **N** | **A** | **N** | **L** | **N** | **G** |  | **E** | **R** |  | **D** | **P** | **V** | **D** | **N** | **R** | **N** | **D** | **D** |  | **N** | **T** | **N** |
| **CHN/km15079/G9P[8]** | **D** | **G** | **S** | **S** | **S** | **N** | **A** | **N** | **L** | **N** | **G** |  | **E** | **R** |  | **D** | **P** | **V** | **D** | **N** | **R** | **N** | **D** | **D** |  | **N** | **T** | **N** |
| **CHN/km15080/G9P[8]** | **D** | **G** | **S** | **S** | **S** | **N** | **A** | **N** | **L** | **N** | **G** |  | **E** | **R** |  | **D** | **P** | **V** | **D** | **N** | **R** | **N** | **D** | **D** |  | **N** | **T** | **N** |
| **CHN/km15081/G9P[8]** | **D** | **G** | **S** | **S** | **S** | **N** | **A** | **N** | **L** | **N** | **G** |  | **E** | **R** |  | **D** | **P** | **V** | **D** | **N** | **R** | **N** | **D** | **D** |  | **N** | **T** | **N** |
| **CHN/km15082/G9P[8]** | **D** | **G** | **S** | **S** | **S** | **N** | **A** | **N** | **L** | **N** | **G** |  | **E** | **R** |  | **D** | **P** | **V** | **D** | **N** | **R** | **N** | **D** | **D** |  | **N** | **T** | **N** |
| **CHN/km15083/G9P[8]** | **D** | **G** | **S** | **S** | **S** | **N** | **A** | **N** | **L** | **N** | **G** |  | **E** | **R** |  | **D** | **P** | **V** | **D** | **N** | **R** | **N** | **D** | **D** |  | **N** | **T** | **N** |
| **CHN/km15084/G9P[8]** | **D** | **G** | **I** | **S** | **S** | **N** | **A** | **N** | **L** | **N** | **G** |  | **E** | **R** |  | **D** | **P** | **V** | **D** | **N** | **R** | **N** | **D** | **D** |  | **N** | **T** | **N** |
| **CHN/km15086/G9P[8]** | **D** | **G** | **S** | **S** | **S** | **N** | **A** | **N** | **L** | **N** | **G** |  | **E** | **R** |  | **D** | **P** | **V** | **D** | **N** | **R** | **N** | **D** | **D** |  | **N** | **T** | **N** |
| **CHN/km15087/G9P[8]** | **D** | **G** | **S** | **S** | **S** | **N** | **A** | **N** | **L** | **N** | **G** |  | **E** | **R** |  | **D** | **P** | **V** | **D** | **N** | **R** | **N** | **D** | **D** |  | **N** | **T** | **N** |
| **CHN/km15088/G9P[8]** | **D** | **G** | **S** | **S** | **S** | **N** | **A** | **N** | **L** | **N** | **G** |  | **E** | **R** |  | **D** | **P** | **V** | **D** | **N** | **R** | **N** | **D** | **D** |  | **N** | **T** | **N** |
| **CHN/km15089/G9P[8]** | **D** | **G** | **S** | **S** | **S** | **N** | **A** | **N** | **L** | **N** | **G** |  | **E** | **R** |  | **D** | **P** | **V** | **D** | **N** | **R** | **N** | **D** | **D** |  | **N** | **T** | **N** |
| **CHN/km15090/G9P[8]** | **D** | **G** | **S** | **S** | **S** | **N** | **A** | **N** | **L** | **N** | **G** |  | **E** | **R** |  | **D** | **P** | **V** | **D** | **N** | **R** | **N** | **D** | **D** |  | **N** | **T** | **N** |
| **CHN/km15091/G9P[8]** | **D** | **G** | **S** | **S** | **S** | **N** | **A** | **N** | **L** | **N** | **G** |  | **E** | **R** |  | **D** | **P** | **V** | **D** | **N** | **R** | **N** | **D** | **D** |  | **N** | **T** | **N** |
| **CHN/km15093/G9P[8]** | **D** | **G** | **S** | **S** | **S** | **N** | **A** | **N** | **L** | **N** | **G** |  | **E** | **R** |  | **D** | **P** | **V** | **D** | **N** | **R** | **N** | **D** | **D** |  | **N** | **T** | **N** |
| **CHN/km15094/G9P[8]** | **D** | **G** | **S** | **S** | **S** | **N** | **A** | **N** | **L** | **N** | **G** |  | **E** | **R** |  | **D** | **P** | **V** | **D** | **N** | **R** | **N** | **D** | **D** |  | **N** | **T** | **N** |
| **CHN/km15095/G9P[8]** | **D** | **G** | **S** | **S** | **S** | **N** | **A** | **N** | **L** | **N** | **G** |  | **E** | **R** |  | **D** | **P** | **V** | **D** | **N** | **R** | **N** | **D** | **D** |  | **N** | **T** | **N** |
| **CHN/km15097/G9P[8]** | **D** | **G** | **S** | **S** | **S** | **N** | **A** | **N** | **L** | **N** | **G** |  | **E** | **R** |  | **D** | **P** | **V** | **D** | **N** | **R** | **N** | **D** | **D** |  | **N** | **T** | **N** |
| **CHN/km15098/G9P[8]** | **D** | **G** | **S** | **S** | **S** | **N** | **A** | **N** | **L** | **N** | **G** |  | **E** | **R** |  | **D** | **P** | **V** | **D** | **N** | **R** | **N** | **D** | **D** |  | **N** | **T** | **N** |
| **CHN/km15099/G9P[8]** | **D** | **G** | **S** | **S** | **S** | **N** | **A** | **N** | **L** | **N** | **G** |  | **E** | **R** |  | **D** | **P** | **V** | **D** | **N** | **R** | **N** | **D** | **D** |  | **N** | **T** | **N** |
| **CHN/km15100/G9P[8]** | **D** | **G** | **S** | **S** | **S** | **N** | **A** | **N** | **L** | **N** | **G** |  | **E** | **R** |  | **D** | **P** | **V** | **D** | **N** | **R** | **N** | **D** | **D** |  | **N** | **T** | **N** |
| **CHN/km15101/G9P[8]** | **D** | **G** | **S** | **S** | **S** | **N** | **A** | **N** | **L** | **N** | **G** |  | **E** | **R** |  | **D** | **P** | **V** | **D** | **N** | **R** | **N** | **D** | **D** |  | **N** | **T** | **N** |
| **CHN/km15102/G9P[8]** | **D** | **G** | **S** | **S** | **S** | **N** | **A** | **N** | **L** | **N** | **G** |  | **E** | **R** |  | **D** | **P** | **V** | **D** | **N** | **R** | **N** | **D** | **D** |  | **N** | **T** | **N** |
| **CHN/km15103/G9P[8]** | **D** | **G** | **S** | **S** | **S** | **N** | **A** | **N** | **L** | **N** | **G** |  | **E** | **R** |  | **D** | **P** | **V** | **D** | **N** | **R** | **N** | **D** | **D** |  | **N** | **T** | **N** |
| **CHN/km15104/G9P[8]** | **D** | **G** | **S** | **S** | **S** | **N** | **A** | **N** | **L** | **N** | **G** |  | **E** | **R** |  | **D** | **P** | **V** | **D** | **N** | **R** | **N** | **D** | **D** |  | **N** | **T** | **N** |
| **CHN/km15105/G9P[8]** | **D** | **G** | **S** | **S** | **S** | **N** | **A** | **N** | **L** | **N** | **G** |  | **E** | **R** |  | **D** | **P** | **V** | **D** | **N** | **R** | **N** | **D** | **D** |  | **N** | **T** | **N** |
| **CHN/km15106/G9P[8]** | **D** | **G** | **S** | **S** | **S** | **N** | **A** | **N** | **L** | **N** | **G** |  | **E** | **R** |  | **D** | **P** | **V** | **D** | **N** | **R** | **N** | **D** | **D** |  | **N** | **T** | **N** |
| **CHN/km15107/G9P[8]** | **D** | **G** | **S** | **S** | **S** | **N** | **A** | **N** | **L** | **N** | **S** |  | **E** | **R** |  | **D** | **P** | **V** | **D** | **N** | **R** | **N** | **D** | **D** |  | **N** | **T** | **N** |
| **CHN/km15108/G9P[8]** | **D** | **G** | **S** | **S** | **S** | **N** | **A** | **N** | **L** | **N** | **G** |  | **E** | **R** |  | **D** | **P** | **V** | **D** | **N** | **R** | **N** | **D** | **D** |  | **N** | **T** | **N** |
| **CHN/km15110/G9P[8]** | **D** | **G** | **S** | **S** | **S** | **N** | **A** | **N** | **L** | **N** | **G** |  | **E** | **R** |  | **D** | **P** | **V** | **D** | **N** | **R** | **N** | **D** | **D** |  | **N** | **T** | **N** |
| **CHN/km15111/G9P[8]** | **D** | **G** | **S** | **S** | **S** | **N** | **A** | **N** | **L** | **N** | **G** |  | **E** | **R** |  | **D** | **P** | **V** | **D** | **N** | **R** | **N** | **D** | **D** |  | **N** | **T** | **N** |
| **CHN/km15113/G9P[8]** | **D** | **G** | **S** | **S** | **S** | **N** | **A** | **N** | **L** | **N** | **G** |  | **E** | **R** |  | **D** | **P** | **V** | **D** | **N** | **R** | **N** | **D** | **D** |  | **N** | **T** | **N** |
| **CHN/km15114/G9P[8]** | **D** | **G** | **S** | **S** | **S** | **N** | **A** | **N** | **L** | **N** | **G** |  | **E** | **R** |  | **D** | **P** | **V** | **D** | **N** | **R** | **N** | **D** | **D** |  | **N** | **T** | **N** |
| **CHN/km15116/G9P[8]** | **D** | **G** | **S** | **S** | **S** | **N** | **A** | **N** | **L** | **N** | **G** |  | **E** | **R** |  | **D** | **P** | **V** | **D** | **N** | **R** | **N** | **D** | **D** |  | **N** | **T** | **N** |
| **CHN/km15118/G9P[8]** | **D** | **G** | **S** | **S** | **S** | **N** | **A** | **N** | **L** | **N** | **G** |  | **E** | **R** |  | **D** | **P** | **V** | **D** | **N** | **G** | **N** | **D** | **D** |  | **N** | **T** | **N** |
